# PD-L1 expression on circulating tumor cells can be a predictive biomarker to PD-1 inhibitors combined with radiotherapy and antiangiogenic therapy in advanced hepatocellular carcinoma

**DOI:** 10.3389/fonc.2022.873830

**Published:** 2022-08-02

**Authors:** Ke Su, Lu Guo, Kun He, Mingyue Rao, Jianwen Zhang, Xiaoli Yang, Weihong Huang, Tao Gu, Ke Xu, Yanlin Liu, Jing Wang, Jiali Chen, Zhenying Wu, Lanxin Hu, Hao Zeng, Hongyan Li, Jian Tong, Xueting Li, Yue Yang, Hanlin Liu, Yaoyang Xu, Zunyuan Tan, Xue Tang, Xunjie Feng, Siyu Chen, Binbin Yang, Hongping Jin, Lechuan Zhu, Bo Li, Yunwei Han

**Affiliations:** ^1^ Department of Oncology, The Affiliated Hospital of Southwest Medical University, Luzhou, China; ^2^ Department of Ophthalmology, The Affiliated Hospital of Southwest Medical University, Luzhou, China; ^3^ Clinical Research Institute, The Affiliated Hospital of Southwest Medical University, Luzhou, China; ^4^ Department of General Surgery (Hepatobiliary Surgery), The Affiliated Hospital of Southwest Medical University, Luzhou, China; ^5^ Nuclear Medicine and Molecular Imaging Key Laboratory of Sichuan Province, Luzhou, China; ^6^ Academician (Expert) Workstation of Sichuan Province, Luzhou, China; ^7^ Clinical Medical College, Southwest Medical University, Luzhou, China; ^8^ Department of Anesthesiology, Affiliated Traditional Chinese Medicine Hospital of Southwest Medical University, Luzhou, China; ^9^ Department of Spinal Surgery, No.1 Orthopedics Hospital of Chengdu, Chengdu, China; ^10^ Department of Oncology, 363 Hospital, Chengdu, China

**Keywords:** programmed death-ligand 1, circulating tumor cells, hepatocellular carcinoma, programmed death 1 inhibitor, radiotherapy, antiangiogenic therapy

## Abstract

**Aim:**

A programmed death 1 (PD-1) inhibitor coupled with radiotherapy and antiangiogenic therapy is a potential therapeutic strategy for advanced hepatocellular carcinoma (HCC). We aimed to determine if circulating tumor cells (CTCs) positive for programmed death-ligand 1 (PD-L1) could be employed as a predictive biomarker in HCC patients receiving triple therapy.

**Methods:**

In this study, HCC patients received a PD-1 inhibitor in combination with intensity-modulated radiotherapy (IMRT) and antiangiogenic therapy. Following IMRT, the PD-1 inhibitor was administrated once every 3 weeks, while the antiangiogenic drug was given once a day. Treatment was continued until the disease progressed. Two mL of peripheral blood was collected at baseline, 1 month, and 3 months after treatment for CTC enrichment using the CytoSorter^®^ system with a CytoSorter™ CTC PD-L1 Kit (Watson Biotech., China).

**Result:**

A total of 47 HCC patients receiving the triple therapy were enrolled in this study. Patients with < 2 PD-L1^+^ CTCs at baseline had a higher objective response rate (ORR) and longer overall survival (OS) than those with ≥ 2 PD-L1^+^ CTCs (56.5% vs. 16.7%, p = 0.007; not reach vs. 10.8 months, p = 0.001, respectively). The count of PD-L1^+^ CTCs was found to be an independent predictive biomarker of OS. Furthermore, the objective response was more likely to be achieved in patients with a dynamic decrease in PD-L1^+^ CTC counts at 1 month after treatment.

**Conclusions:**

Our study demonstrated that PD-L1^+^ CTCs could be a predictive biomarker for HCC patients receiving PD-1 inhibitors in combination with IMRT and antiangiogenic therapy.

## Introduction

Hepatocellular carcinoma (HCC) is a highly invasive cancer and has a median overall survival (mOS) of 2–4 months (1, 2). Recently, programmed death 1 (PD-1)/programmed death-ligand 1 (PD-L1) inhibitors opened up new perspectives in the therapy of advanced HCC patients (3, 4). However, their efficacy alone is limited in advanced HCC patients.

The combination therapy of PD-1/PD-L1 inhibitors with antiangiogenic drugs, such as pembrolizumab plus lenvatinib (5), sintilimab plus bevacizumab (6), and camrelizumab plus apatinib (7), attained good clinical results in advanced HCC patients and improved overall survival (OS) over single-agent therapy. At the same time, nivolumab combined with radiotherapy had also demonstrated significant efficacy in advanced HCC (8). In addition, Zhong et al. found that PD-1/PD-L1 inhibitors in combination with radiotherapy and antiangiogenic therapy could improve the mOS to 22.4 months and the objective response rate (ORR) to 40% with low toxicity in advanced HCC patients (9). Radiotherapy and antiangiogenic agents can not only promote T cell infiltration into tumors, but they can also improve responsiveness to immunotherapy (10–12). As a result, based on these findings, triple therapy appears to be a safe and promising treatment strategy for HCC. However, predicting the efficacy of triple therapy remains a challenge.

The PD-L1 expression in a tumor is an important factor in the cancer immune cycle (13). The binding of PD-L1/PD-1 could prompt immune evasion and suppress T cell activation (14, 15). In the current studies, the PD-L1 expression could be detected using tissue biopsy and liquid biopsy (circulating tumor cells, CTCs) (13, 16). However, because the spatial-temporal heterogeneity of PD-L1 expression is inevitable, the information needed for the selection of appropriate therapy is limited to a single biopsy at a specific point (17–19). CTCs exhibit the advantages of continuous assessment, low invasiveness, and interrogation of the overall tumor burden, rather than a specific area (20, 21). Therefore, CTCs appear to be a more promising technique to assess the dynamic expression of PD-L1 in tumors and offer a better understanding of tumor heterogeneity compared to tissue biopsy.

Currently, multiple studies showed that PD-L1^+^ CTCs could be a prognostic indicator in a variety of cancers including HCC (22, 23). However, there was a controversy about the link between PD-L1^+^ CTCs and clinical efficacy (21, 24–26). The triple therapy had improved the prognosis of HCC patients significantly (9). We aimed to confirm whether PD-L1^+^ CTCs could be a predictive biomarker in HCC patients treated with triple therapy.

## Materials and methods

### Study design and participants

In this study, 47 patients received a PD-1 inhibitor combined with intensity-modulated radiotherapy (IMRT) and antiangiogenic therapy, followed by a PD-1 inhibitor injection once every three weeks as well as the antiangiogenic drug on a daily basis until the appearance of intolerable toxic reactions or progressive disease (PD). From the first day of triple therapy, the PD-1 inhibitor and the antiangiogenic drug were given. We investigated the dynamic changes in CTC and PD-L1^+^ CTC counts at 3 days before initiating PD-1 inhibitors (T0), 1 month (T1), and 3 months (T2) after starting triple therapy.

The inclusion criteria were as follows: (a) age exceeded 18 years; (b) HCC was diagnosed by histology or cytology; (c) Eastern Cooperative Oncology Group performance status (ECOG PS) score of 0/1; (d) Barcelona Clinic Liver Cancer (BCLC) stage B/C; and (e) expected survival time exceeded 3 months.

The exclusion criteria were as follows: (a) history of liver transplantation, esophageal variceal bleeding, autoimmune disease, massive ascites, hepatic encephalopathy, and abdominal infection; (b) tumors affecting adjacent organs or have a residual liver volume of <700 ml, which are difficult to treat with radiotherapy; (c) white blood cell (WBC) counts > 10 × 10^9^/L; and (d) time of processing of blood samples was more than 6 hours after collection.

This trial complied with the Declaration of Helsinki and was approved by the Clinical Trials Ethics Committee of the Affiliated Hospital of Southwest Medical University (approval number KY2021063). Written informed consent was obtained from each patient. The study was registered at the Chinese Clinical Trials Registry: ChiCTR2100044198.

### CTC enrichment and PD-L1^+^ CTC enumeration

The CytoSorter™ BioScanner system (Watson Biotech., China) was used for the CTC test (27). Briefly, 2 mL of collected peripheral blood was processed within 6h at room temperature and mixed with PBS in a ratio of 1:1, followed by density gradient centrifugation (1000g × 10min). Peripheral blood mononuclear cells (PBMCs) were acquired *via* centrifugation. The PBMC fraction was then washed and then transferred into the CytoSorter™ BioScanner system. Then, a CytoSorter™ CTC PD-L1 Kit (Watson Biotech., China) was used to identify CTCs. CTCs were captured using a microfluidic chip immobilized with cell surface vimentin (CSV) and epithelial cell adhesion molecules (EpCAM), followed by immunofluorescence staining with Pan-cytokeratin (CK)-fluorescein isothiocyanateand (FITC), CSV-FITC, CD45-lymphocyteantigen-phycoerythrin (PE), and PD-L1-Cyanine 5 (Cy5), and Hoechst33342 was used for nuclear staining. An automated fluorescence microscope equipment (Nikon Ti-E) was used to verify the localization and staining of PD-L1^+^ CTCs. CTCs were defined as CSV&CK^+^, CD45, and Hoechst^+^.

### Efficacy

Treatment response was determined by comparing imaging examinations (computed tomography or magnetic resonance imaging) every 6-8 weeks to the baseline imaging examination. According to Response Evaluation Criteria in modified Solid Tumors (mRECIST), patients were divided into nonresponders (PD and stable disease (SD)) and responders (partial response (PR) and complete response (CR)). The date from the start of triple therapy to the last follow-up or death was used to calculate OS. The date from the start of triple therapy to PD was used to calculate progression-free survival (PFS).

### Statistical Analysis

Continuous variables were analyzed using the Mann-Whitney U and Wilcoxon matched-pairs signed-rank tests. Categorical variables were analyzed using the *χ2* test. The best cutoff value for distinguishing nonresponders from responders was determined by the receiver operating characteristic (ROC) curve. Then, the relationship between PD-L1^+^ CTCs (high vs. low) and ORR was assessed by the univariate logistic regression model. PFS and OS were estimated using Kaplan–Meier statistics. We introduced variables with P < 0.05 confirmed in univariate analysis into multivariate analysis to identify independent prognostic factors for PFS and OS. SPSS for Windows (version 26.0) was utilized for all statistical analyses. Two-tailed P-values < 0.05 were considered statistically significant.

## Result

### Patient characteristics

**Figure 1:**
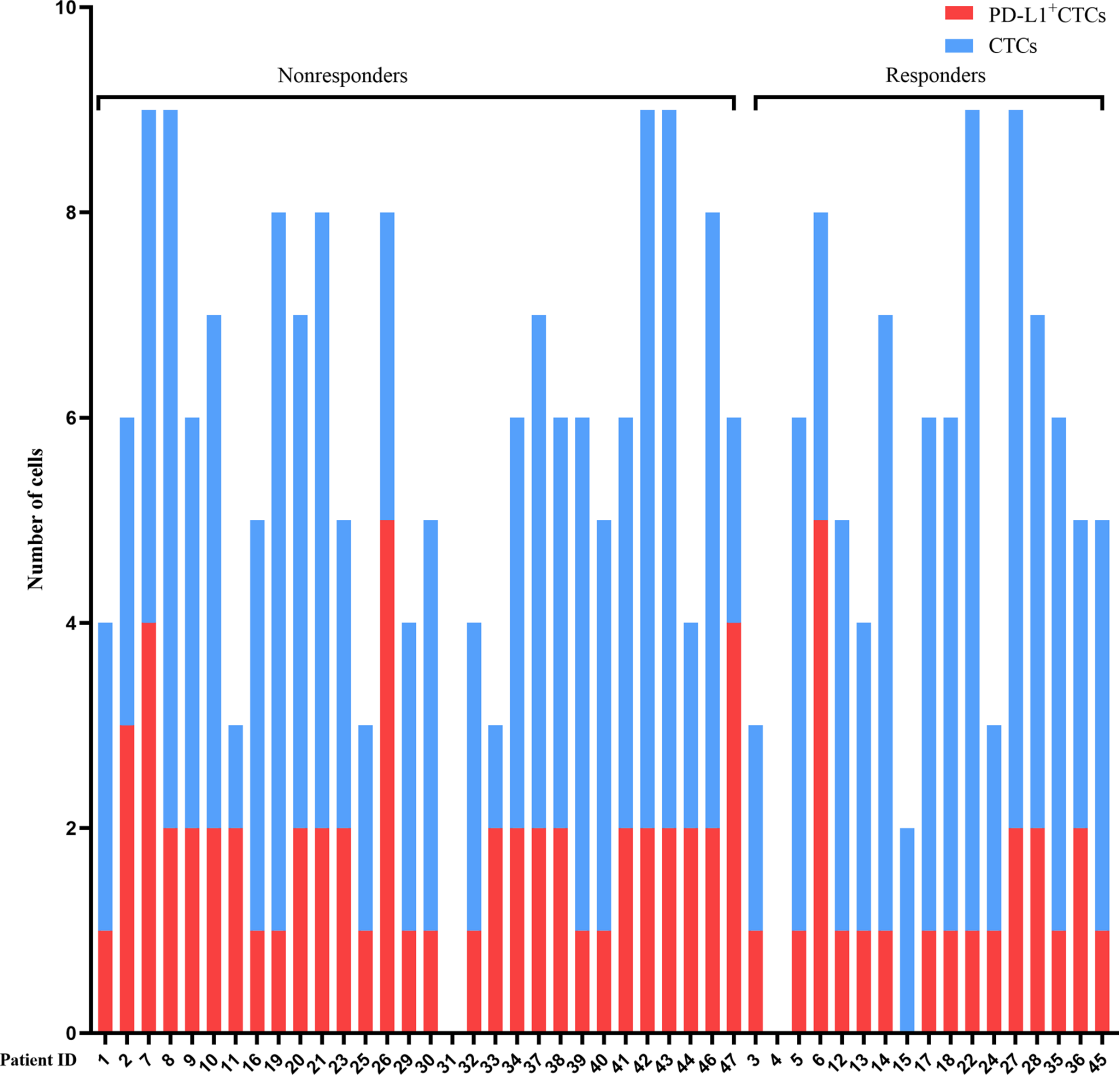
Detection of CTCs and PD-L1^+^ CTCs. The counts of CTCs (blue bars) and PD-L1^+^ CTCs (red bars) per patient detected are presented at baseline. Nonresponders and responders were divided into two groups. PD-L1, programmed death-ligand 1; CTCs, circulating tumor cells.

In this study, a total of 47 HCC patients receiving triple therapy had experienced at least one CTC test between June 1, 2019, and October 30, 2021. The median PD-L1^+^ CTC count was 2 (range 0–5). All baseline characteristics are shown in **Table 1** and **Figure 1**. The median course of the PD-1 inhibitor was 6 (range 2–22). The number of patients receiving the various types of PD-1 inhibitors were as follows: sintilimab (n = 10), camrelizumab (n = 11), and tislelizumab (n = 26). All patients received concurrent antiangiogenic agents, including apatinib (n = 1), regorafenib (n = 2), sorafenib (n = 8), anlotinib (n = 15), and lenvatinib (n = 21) (**Supplementary Table 1**). All patients received IMRT and the target total dose was 48 Gy delivered in 3 Gy/fraction.

**Table 1:** Baseline characteristics.

Variable	Total	< 2 PD-L1^+^ CTCs	≥ 2 PD-L1^+^ CTCs	p value
	(n = 47)	(n = 23)	(n = 24)	
Male sex	45 (95.7)	23 (100.0)	22 (91.7)	0.157
Age ≥ 60 years	13 (27.7)	9 (39.1)	4 (16.7)	0.085
Child–Pugh B	13 (27.7)	4 (17.4)	9 (37.5)	0.123
Number of tumors ≥ 2	34 (72.3)	14 (60.9)	20 (83.3)	0.085
Tumor size, median	7.4 (1.6–22.8)	7.5 (2.6–15.0)	7.2 (1.6–22.8)	0.79
(range, ng/ml)				
< 5 cm	8 (17.0)	5 (21.7)	3 (12.5)	
≥ 5 cm	39 (83.0)	18 (78.3)	21 (87.5)	
Serum AFP, median	202.3 (1.0–432408.8)	55.2 (2.3–73477.1)	593.7 (1.0–432408.8)	0.419
(range, ng/ml)				
< 400 ng/ml	25 (53.2)	14 (60.9)	11 (45.8)	
≥ 400 ng/ml	22 (46.8)	9 (39.1)	13 (54.2)	
ECOG PS				0.018
0	22 (46.8)	15 (65.2)	7 (29.2)	
1	21 (44.7)	8 (34.8)	13 (54.2)	
2	4 (8.5)	0	4 (16.7)	
CTCs counts,	6 (0–9)	5 (0–9)	7 (3–9)	
median (range, ng/ml)				
PD-L1^+^ CTCs counts,	2 (0–5)	`1 (0–1)	2 (2–5)	
median (range, ng/ml)				
BCLC stage				0.157
B	2 (4.3)	0	2 (8.3)	
C	45 (95.7)	23 (100)	22 (91.7)	
Portal vein invasion	42 (89.4)	22 (95.7)	20 (83.3)	0.171
Etiology				
HBV	32 (68.1)	17 (73.9)	15 (62.5)	0.401
HCV	2 (4.3)	1 (4.3)	1 (4.2)	0.975
Alcohol	14 (29.8)	5 (21.7)	9 (37.5)	0.238
Lymph node metastasis	20 (42.6)	9 (39.1)	11 (45.8)	0.642
Extrahepatic metastases	10 (21.3)	6 (26.1)	4 (16.7)	0.43
Lung	4 (8.5)	3 (13.0)	1 (4.2)	
Bone	6 (12.8)	3 (13.0)	3 (12.5)	
Previous therapy	23 (48.9)	11 (47.8)	12 (50.0)	0.882
Systemic therapy	15 (31.9)	7 (30.4)	8 (33.3)	0.831
Liver resection	6 (12.8)	4 (17.4)	2 (8.3)	0.352
radiotherapy	4 (8.5)	2 (8.7)	2 (8.3)	0.965
TACE	16 (34.0)	8 (34.8)	8 (33.3)	0.917
RFA	4 (8.5)	1 (4.3)	3 (12.5)	0.317

AFP, alpha fetoprotein; ECOG PS, Eastern Cooperative Oncology Group performance status; PD-L1, programmed death-ligand 1; CTCs, circulating tumor cells; BCLC, barcelona clinic liver cancer; HBV, hepatitis B virus; HCV, hepatitis C virus; TACE, transcatheter arterial chemoembolization; RFA, radiofrequency ablation.

At the data deadline (December 9, 2021), the median follow-up duration was 9.2 months (95% CI 5.7–12.8), 15 (31.9%) patients were dead, and 10 (58.8% of responders) patients had ongoing responses. A median PFS (mPFS) of 8.6 months (95% CI 6.0–11.2), an mOS of 20.1 months (95% CI 11.6–28.6), an ORR of 36.1%, and a disease control rate (DCR = CR + PR + SD) of 91.5% were obtained in the total cohort (**Table 2**). CTCs and PD-L1^+^ CTCs were confirmed to exist in 45 (95.7%) and 44 (93.6%) patients, respectively. In a subgroup of tislelizumab in combination with IMRT and lenvatinib (n = 14), the mOS was 20.3 months and the mPFS was 10.2 months.

**Table 2:** Antitumor activity of patients with ≥ 2 or < 2 PD-L1^+^ CTCs assessed by mRECIST .

Antitumor Activity	Total	< 2 PD-L1^+^ CTCs	≥ 2 PD-L1^+^ CTCs
	(n = 47), n (%)	(n = 23), n (%)	(n = 24), n (%)
Objective response	17 (36.1)	13 (56.5)	4 (16.7)
Disease control rate	43 (91.5)	22 (96.7)	21 (87.5)
Best overall response			
Complete response	1(2.1)	1 (4.3)	0
Partial response	16 (34.0)	12 (52.2)	4 (16.7)
Stable disease	26 (55.3)	9 (39.1)	17 (70.8)
Progressive disease	4 (8.5)	1 (4.3)	3 (12.5)

PD-L1, programmed death-ligand 1; CTCs, circulating tumor cells; mRECIST, Response Evaluation Criteria in modified Solid Tumors.

### PD-L1^+^ CTCs at T0 and response to triple therapy

**Figure 2:**
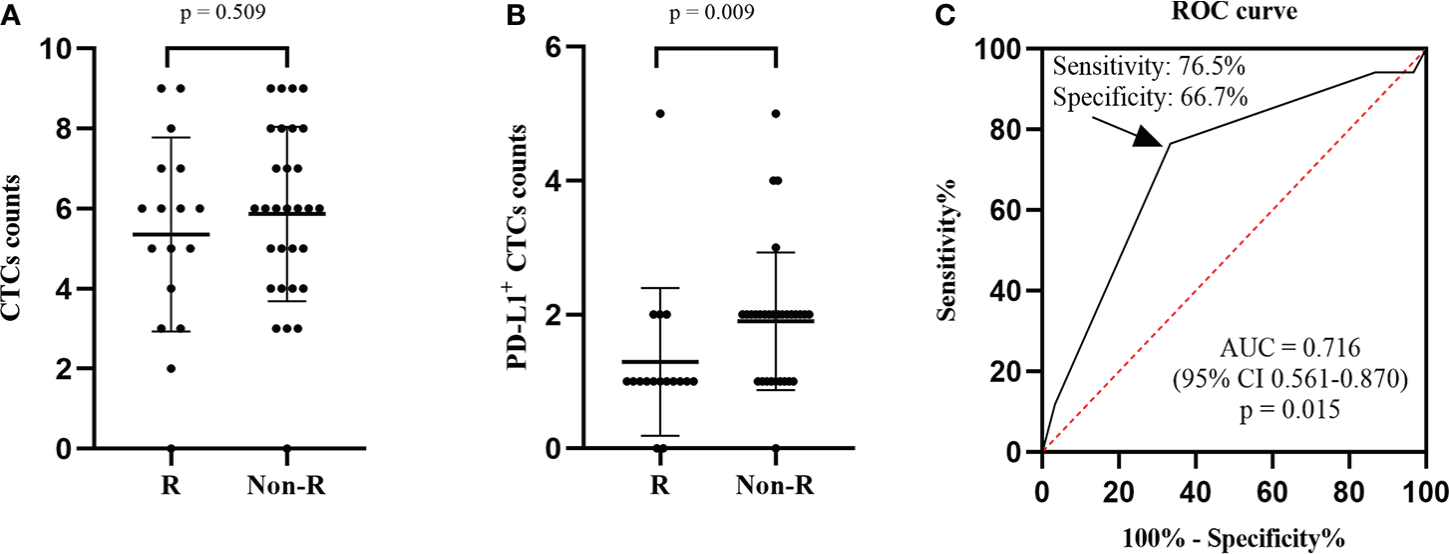
Comparison of the number of CTCs **(A)** and PD-L1^+^ CTCs **(B)** between the nonresponse group and response group. **(C)** ROC curve was adopted to investigate the predictive value of PD-L1^+^ CTC counts. When applying a cut-off of 2 PD-L1^+^ CTCs, a specificity of 65.5%, a sensitivity of 76.5%, and an AUC of 0.710 were observed. R, responder; CTCs, circulating tumor cells; PD-L1, programmed death-ligand 1; ROC, receiver operating characteristic; CI, confidence interval; AUC, area under the curve.

The ORR of 36.1% (17 of 47) was obtained in our cohort. We found that the CTC counts of nonresponders and responders were not statistically different at baseline (p = 0.509, **Figure 2A**). However, the nonresponders had higher PD-L1+ CTC counts than the responders (p = 0.009, **Figure 2B**). At the same time, the predictive value of PD-L1^+^ CTC counts was investigated using the ROC curve. We found that the area under the curve (AUC) was 0.716 and a cut-off value of 2 PD-L1^+^ CTCs had the biggest Youden index of 0.43. When a cut-off value of 2 PD-L1^+^ CTCs was applied, a specificity of 66.7% and a sensitivity of 76.5% were observed (**Figure 2C**). In addition, in the univariate logistic regression model, the response of patients with < 2 PD-L1^+^ CTCs was 6.5 times that of patients with ≥ 2 PD-L1^+^ CTCs (OR 6.500, 95% CI 1.679-25.162, p = 0.007, **Table 2**).

### Prognostic significance of PD-L1^+^ CTCs at T0

**Figure 3:**
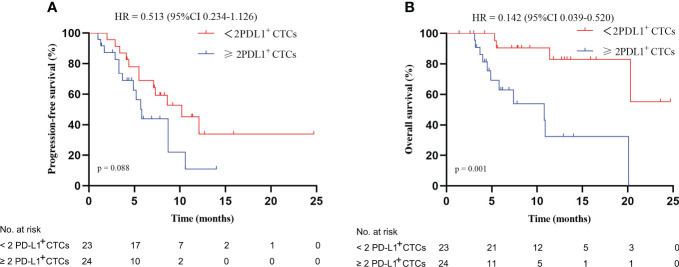
Kaplan–Meier plots: **(A)** progression-free survival and **(B)** overall survival based on PD-L1 expression on CTCs at baseline. HR, hazard ratio; CI, confidence interval; CTCs, circulating tumor cells; PD-L1, programmed death-ligand 1.

**Figure 4:**
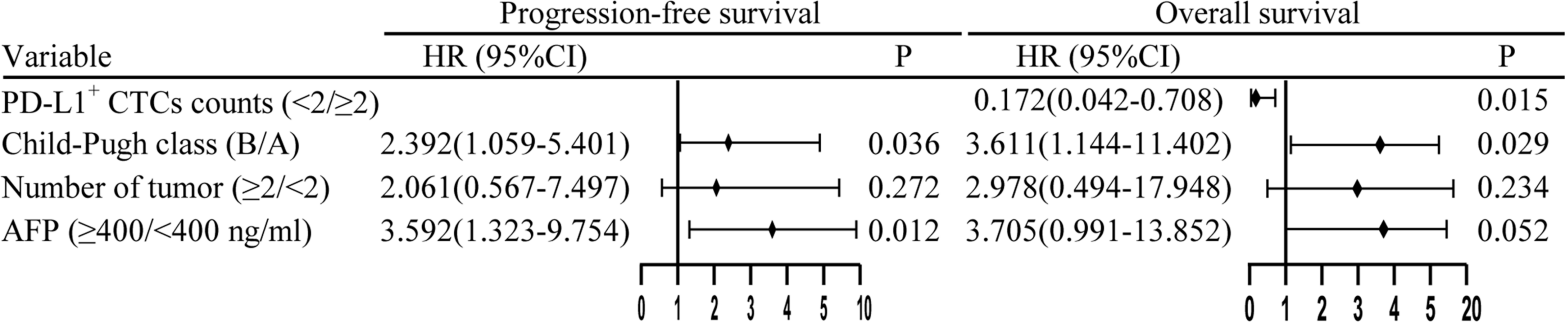
Multivariate Cox regression analysis of progression-free survival and overall survival. HR, hazard ratio; CI, confidence interval; AFP, alpha fetoprotein; PD-L1, programmed death-ligand 1; CTCs, circulating tumor cells.

In this study, patients with < 2 PD-L1^+^ CTCs at T0 did not have a better PFS than patients with ≥ 2 PD-L1^+^ CTCs (p = 0.088, **Figure 3A**). However, patients with < 2 PD-L1^+^ CTCs had a better mOS than patients with ≥ 2 PD-L1^+^ CTCs (not reach vs. 10.8 months [95% CI 3.8–17.8], p = 0.001, **Figure 3B**), with a HR of 0.142 (95% CI 0.039–0.520). In the univariate analysis, we demonstrated that lower PD-L1^+^ CTCs were associated with a better OS (HR, 0.142, 95% CI, 0.039–0.520, p = 0.003, **Supplementary Table 2**). In the multivariate analysis, < 2 PD-L1^+^ CTCs were identified as a positive independent prognostic factor for OS (HR 0.172, 95% CI 0.042–0.708, p = 0.015; **Figure 4**).

### PD-L1^+^ CTCs at T0 correlate with the characteristics of patients

In order to evaluate the link between PD-L1^+^ CTCs and the clinical characteristics of patients, patients with ≥ 2 and < 2 PD-L1^+^ CTCs were divided into two groups (**Table 1**). There were no differences in the BCLC stage, AFP levels, and sex between the two groups. However, patients with ≥ 2 PD-L1^+^ CTCs had worse ECOG PS than those with < 2 PD-L1^+^ CTCs.

### Change of CTC and PD-L1^+^ CTC counts after triple therapy

**Figure 5:**
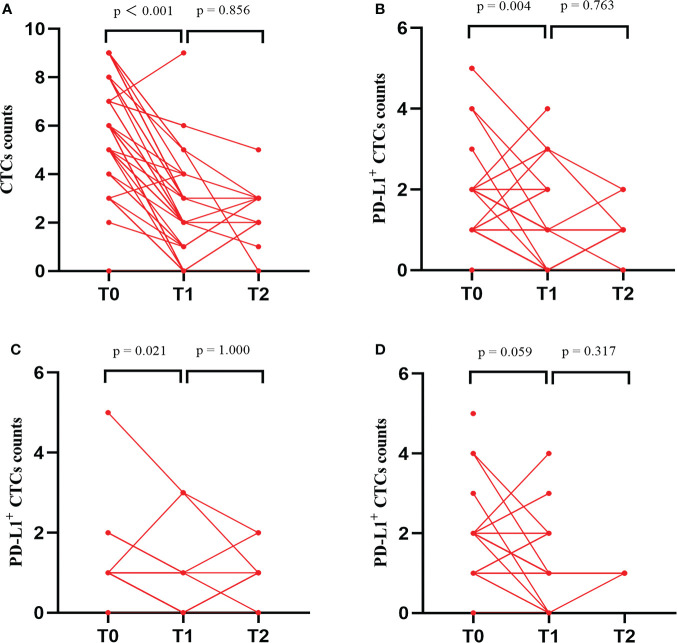
Changes in total CTC counts, PD-L1^+^ CTC counts and proportion of PD-L1^+^ CTC counts at baseline (T0), 1 months (T1), and 3 months (T2) after the beginning of triple therapy. **(A)** CTC counts in total enrolled patients. **(B)** PD-L1^+^ CTC counts in total enrolled patients. **(C)** PD-L1^+^ CTC counts in responders. **(D)** PD-L1^+^ CTC counts in nonresponders. PD-L1, programmed death-ligand 1; CTCs, circulating tumor cells.

A total of 47, 42, and 14 HCC patients had completed the CTC test at T0, T1, and T2, respectively, after the patient’s consent. At T1, we found that the CTC counts in all enrolled patients (**Figure 5A**), PD-L1^+^ CTC counts in all enrolled patients (**Figure 5B**), and PD-L1^+^ CTC counts in responders (**Figure 5C**) decreased upon treatment, but the PD-L1^+^ CTC counts in nonresponders (**Figure 5D**) remained unchanged. In addition, a comparison of the CTC test at T1 and T2 illustrated that no change was observed in all of the above elements. The details of the PD-L1^+^ CTC counts are shown in **Supplementary Table 3**.

### Prognostic significance of PD-L1^+^ CTCs at T1

One or fewer PD-L1^+^ CTCs at T1 were not associated with a better ORR, according to the univariate logistic regression model (HR 1.926, 95% CI 0.427–8.688, p = 0.394). Patients with < 2 PD-L1^+^ CTCs had a similar mPFS and mOS compared to patients with ≥ 2 PD-L1^+^ CTCs (8.6 vs. 4.9 months, p = 0.156, **Supplementary Figure 1A**; 20.6 vs. 7.4 months, p = 0.056, **Supplementary Figure 1B**; respectively).

### Contribution of the tumor size and metastases to the number of PD-L1^+^ CTCs and the clinical response

In the univariate logistic regression model, the tumor size, lymph node metastasis, and extrahepatic metastasis were not correlated with the number of PD-L1^+^ CTCs (OR 0.514, 95% CI 0.108-2.456, P = 0.405; OR 0.760, 95% CI 0.238-2.424, P = 0.642; OR 1.765, 95% CI 0.426-7.307, P = 0.433, respectively) and ORR (OR 0.500, 95% CI 0.107-2.327, P = 0.377; OR 0.623, 95% CI 0.183-2.125, P = 0.450; OR 0.367, 95% CI 0.068-1.973, P = 0.243, respectively).

## Discussion

In our study, we performed multiple assessments on the PD-L1 expression on CTCs of HCC patients treated with triple therapy using the CytoSorter™ BioScanner system. The sensitivity and specificity of this approach were clinically validated in different solid cancers including HCC, breast cancer, lung cancer, and pancreatic cancer, with 61.9%-95.4% sensitivity and 76.6%-91.3% specificity (27–30). Our data showed that HCC patients with < 2 PD-L1^+^ CTCs were related to a better OS and ORR than those with ≥ 2 PD-L1^+^ CTCs. Moreover, we demonstrated that the decrease of PD-L1^+^ CTC counts at T1 after the beginning of triple therapy was significantly related to the ORR, which proved the necessity of continuous assessment of PD-L1^+^ CTCs.

The mOS of advanced HCC patients was only 2–4 months (1, 2). The emergence of PD-1/PD-L1 inhibitors completely revolutionized the treatment for advanced HCC patients. Although patients in our study had a poor tumor burden, the ORR of 36.1%, DCR of 91.5%, mPFS of 8.6 months, and mOS of 20.1 months were encouraging.

Currently, many biomarkers such as alpha fetoprotein (AFP), alkaline phosphatase (ALP), and PD-L1 expression had been assessed in previous studies with promising outcomes, particularly PD-L1 expression that had been proven to be linked to the ORR of PD-1/PD-L1 inhibitors in various cancers including HCC (21, 22, 31, 32). The triple therapy improved the prognosis of HCC patients significantly (9), although the correlation between PD-L1^+^ CTCs and their prognosis remains unclear. In a meta-analysis of patients with cancer receiving immune checkpoint inhibitors, the expression of PD-L1 on CTC was not found to be associated with the patient’s prognosis (20). However, Sonja et al. demonstrated that urothelial carcinoma patients receiving immunotherapy with < 2 PD-L1^+^ CTCs had a better OS than those with ≥ 2 PD-L1^+^ CTCs (p = 0.008) (23). In contrast, in a report of pembrolizumab in melanoma by Khattak et al., the presence of PD-L1^+^ CTC was associated with longer PFS and OS (33). Ikeda et al. also reported the association of PD-L1^+^ CTC with longer PFS in lung cancer patients receiving nivolumab (21). Moreover, in a study of HCC patients receiving PD-1/PD-L1 inhibitors, patients without PD-L1^+^ CTCs had a worse ORR than patients with PD-L1^+^ CTCs (22). It did not further explore the predictive value of a specific number of PD-L1^+^ CTCs. In this study, we found that a cut-off value of 2 PD-L1+ CTCs had the highest capability of prediction for ORR and patients with < 2 PD-L1+ CTCs were related to a better ORR and mOS. Thence, the PD-L1+ CTCs may be related to the mechanism of tumor escape (34).

Subsequently, we conducted multivariate Cox regression analysis and found that PD-L1^+^ CTCs were proven to be an independent predictive biomarker factor of OS. Furthermore, we investigated the dynamic change in PD-L1^+^ CTC counts and noted that the PD-L1^+^ CTC counts declined at T1 in the responder group and increased in the nonresponder group (p < 0.05). This revealed that both < 2 PD-L1^+^ CTCs at T0 and dynamic decrease in PD-L1^+^ CTC counts at T1 could be used to assess prognosis. However, we found that one or less PD-L1^+^ CTCs at T1 were not related to a better ORR, mPFS, and mOS. These findings may be resulting from the small sample size (n = 47). In addition, the PD-L1^+^ CTCs at T2 remained unchanged in both responders and nonresponders upon treatment (p > 0.05). This indicated that the unchanged PD-L1^+^ CTC counts at the later stage of triple therapy were not a predictor of efficacy but rather a maintenance of long-term efficacy.

There are currently no biomarkers that can be used to guide the use of PD-1 inhibitors in combination with radiation and antiangiogenic therapy and reflect therapeutic response. These findings highlight the importance of dynamic evaluation of PD-L1 expression in identifying HCC patients who are suitable for triple therapy and supporting physicians to alter treatments as soon as possible for HCC patients who are unlikely to respond.

Of course, there were certain limitations to the study. An important limitation of the study was the use of different antiangiogenic agents as well as different PD-1 inhibitors. Substantial heterogeneity in the treatment regimen might have influenced the interpretation of our findings. In addition, this was a small sample, single-arm trial, and not all of the patients completed the CTC test at T1 and T2. As a result, bigger sample size is required to determine whether these findings and the ROC curve cutoff values are solely appropriate for present patients.

## Conclusions

In conclusion, our study showed that HCC patients with < 2 PD-L1^+^ CTCs at baseline had a better ORR and OS receiving triple therapy. Also, < 2 PD-L1^+^ CTCs were a positive independent prognostic factor for OS. Furthermore, this study demonstrated that the response was more likely to be achieved in patients with a dynamic decrease in PD-L1^+^ CTC counts at 1 month. These results support PD-L1^+^ CTCs as a predictive biomarker for HCC patients and provide a benchmark for future trials.

## Data availability statement

The original contributions presented in the study are included in the article/**Supplementary Material**. Further inquiries can be directed to the corresponding authors.

## Ethics statement

The studies involving human participants were reviewed and approved by Clinical Trials Ethics Committee of the Affiliated Hospital of Southwest Medical University. The patients/participants provided their written informed consent to participate in this study.

## Author contributions

KS, LG, KH, MR, JZ, XY, WH, TG, KX, YL, JW, JC, ZW, LH, HZ, HYL, JT, XL, YY, HLL, YX, ZT, XT, XF, SC, BY, HJ, and LZ collected the data. YH and BL designed the research study. KS, LG, KH, BL and YH wrote the manuscript and analyzed the data. All authors approved the final version of the manuscript. All authors contributed to the article and approved the submitted version.

## Funding

This work was supported by a grant from Project of Science and Technology Department of Sichuan Province (2020JDTD0036).

## Acknowledgments

The authors thank Hangzhou Watson Biotech, Inc. (Hangzhou, China) for technical help in CTC detection. The authors also thank the patients and families who made this study possible and the investigators and the clinical study teams who participated in this study.

## Conflict of interest

The authors declare that the research was conducted in the absence of any commercial or financial relationships that could be construed as a potential conflict of interest.

## Publisher’s note

All claims expressed in this article are solely those of the authors and do not necessarily represent those of their affiliated organizations, or those of the publisher, the editors and the reviewers. Any product that may be evaluated in this article, or claim that may be made by its manufacturer, is not guaranteed or endorsed by the publisher.
