# Supplementary material for: PD-L1 expression on circulating tumor cells can be a predictive biomarker to PD-1 inhibitors combined with radiotherapy and antiangiogenic therapy in advanced hepatocellular carcinoma
Source: Front Oncol. 2022 Aug 2;12:873830. doi: 10.3389/fonc.2022.873830 (PMC9379259; doi:10.3389/fonc.2022.873830)
Supplement: Supplementary file 1 [file DataSheet_1.pdf]

Supplementary Table 1. Treatment details, detailed characteristics and outcome of patients (n = 47).

ID	PD-1 inhibitor (course)	Targeted agent	OS months	Response	Status
1	Tislelizumab (14)	Lenvatinib	20.3	SD	Death
2	Tislelizumab (3)	Anlotinib	4.5	PD	Death
3	Sintilimab (9)	Anlotinib	11.4	PR	Death
4	Sintilimab (8)	Lenvatinib	23.6	PR	Alive
5	Tislelizumab (21)	Lenvatinib	15.9	CR	Alive
6	Tislelizumab (4)	Anlotinib	7.4	PR	Death
7	Tislelizumab (2)	Sorafenib	3.1	PD	Death
8	Tislelizumab (13)	Lenvatinib	12.9	SD	Alive
9	Tislelizumab (3)	Lenvatinib	5.8	SD	Death
10	Camrelizumab (3)	Anlotinib	4.9	SD	Death
11	Sintilimab (6)	Anlotinib	20.1	SD	Death
12	Sintilimab (6)	Sorafenib	7.8	PR	Alive
13	Tislelizumab (15)	Lenvatinib	11.4	PR	Alive
14	Tislelizumab (11)	Anlotinib	13.3	PR	Alive
15	Tislelizumab (10)	Lenvatinib	13	PR	Alive
16	Tislelizumab (4)	Anlotinib	5.5	SD	Death
17	Tislelizumab (7)	Lenvatinib	12.7	PR	Alive
18	Tislelizumab (9)	Sorafenib	11.8	PR	Alive
19	Sintilimab (8)	Sorafenib	9.2	SD	Alive
20	Tislelizumab (2)	Lenvatinib	3.2	PD	Death
21	Tislelizumab (3)	Lenvatinib	3.7	SD	Death
22	Sintilimab (5)	Lenvatinib	8.3	PR	Alive
23	Camrelizumab (6)	Anlotinib	6.2	SD	Alive
24	Tislelizumab (7)	Lenvatinib	7.9	PR	Alive
25	Tislelizumab (4)	Lenvatinib	7.9	SD	Alive
26	Sintilimab (5)	Anlotinib	7.8	SD	Alive
27	Tislelizumab (3)	Anlotinib	4	PR	Death
28	Camrelizumab (5)	Anlotinib	10.8	PR	Death
29	Tislelizumab (4)	Lenvatinib	16.5	SD	Alive
30	Sintilimab (3)	Anlotinib	5.3	SD	Death
31	Tislelizumab (22)	Lenvatinib	24.7	SD	Alive
32	Camrelizumab (3)	Lenvatinib	13.7	SD	Alive
33	Tislelizumab (9)	Sorafenib	14	SD	Alive
34	Tislelizumab (7)	Sorafenib	10.9	SD	Death
35	Tislelizumab (7)	Anlotinib	7.2	PR	Alive
36	Tislelizumab (8)	Sorafenib	6.9	PR	Alive
37	Camrelizumab (3)	Lenvatinib	3.3	SD	Alive
38	Camrelizumab (9)	Regorafenib	6	SD	Alive
39	Camrelizumab (2)	Apatinib	3	PD	Alive
40	Tislelizumab (7)	Anlotinib	5.6	SD	Alive
41	Tislelizumab (5)	Lenvatinib	4.7	SD	Alive

42	Camrelizumab (4)	Lenvatinib	4.4	SD	Alive
43	Camrelizumab (5)	Anlotinib	4.2	SD	Alive
44	Sintilimab (6)	Lenvatinib	4.2	SD	Alive
45	Sintilimab (6)	Sorafenib	4.2	PR	Alive
46	Camrelizumab (3)	Lenvatinib	2.6	SD	Alive
47	Camrelizumab (2)	Regorafenib	1.4	SD	Alive

Abbreviations: PD-1, programmed death 1; OS, overall survival; CR, complete response; PR, partial response; SD, stable disease; PD, progressive disease.

Supplementary Table 2. Univariate Cox regression analysis of progression-free survival and overall survival

	Progression-free survival			Overall survival		
Variable	HR	95%CI	*P*	HR	95%CI	*P*
Sex (female/male)	0.043	0.001-27.007	0.338	0.045	0.001-1177.192	0.551
Age (≥60/<60 years)	0.458	0.181-1.160	0.100	0.319	0.072-1.418	0.133
Child-Pugh class (B/A)	3.018	1.369-6.657	0.006	5.135	1.717-15.362	0.003
Number of tumor (≥2/<2)	5.180	1.648-16.277	0.005	5.780	1.251-26.708	0.025
Tumor size (≥5/<5 cm)	1.115	0.383-3.242	0.842	0.774	0.213-2.817	0.698
AFP (≥400/<400 ng/ml)	5.109	2.087-12.51	< 0.001	3.789	1.283-11.188	0.016
ECOG PS (1-2/0)	1.386	0.628-3.060	0.419	1.542	0.518-4.595	0.437
PD-L1^{+} CTC counts (<2/≥2)	0.513	0.234-1.126	0.096	0.142	0.039-0.520	0.003
BCLC stage (B/C)	0.046	0.001-5792.205	0.608	0.046	0.001-8470444.981	0.752
Portal vein invasion (no/yes)	0.639	0.084-4.846	0.665	0.044	0.001-6856.645	0.609
HBV (positive/negative)	1.275	0.508-3.200	0.605	1.016	0.275-3.755	0.981
HCV (positive/negative)	1.143	0.268-4.878	0.857	1.041	0.134-8.119	0.969
Alcoholism (positive/negative)	1.755	0.793-3.883	0.165	1.510	0.533-4.277	0.438
Lymph node metastasis (yes/no)	1.817	0.833-3.966	0.134	2.318	0.788-6.820	0.127
Extrahepatic metastases (yes/no)	1.482	0.618-3.553	0.378	0.678	0.180-2.550	0.565
Previous therapy (yes/no)	0.713	0.326-1.560	0.397	0.487	0.158-1.503	0.210

Abbreviations: HR, hazard ratio; CI, confidence interval; AFP, alpha fetoprotein; ECOG PS, Eastern Cooperative Oncology Group performance status; PD-L1, programmed death-ligand 1; CTCs, circulating tumor cells; BCLC, Barcelona Clinic Liver Cancer; HBV, hepatitis B virus; HCV, hepatitis C virus;

Supplemental Table 3. Details of CTCs and PD-L1$^+$ CTCs counts in the all patients

ID	T0		T1		T2		ORR
	CTC counts	PD-L1$^+$ CTC counts	CTC counts	PD-L1$^+$ CTC counts	CTC counts	PD-L1$^+$ CTC counts	
1	4	1	2	1	-	-	No
2	6	3	0	0	-	-	No
3	3	1	0	0	-	-	Yes
4	0	0	-	-	-	-	Yes
5	6	1	0	0	-	-	Yes
6	8	5	5	3	3	1	Yes
7	9	4	3	2	-	-	No
8	9	2	3	1	3	1	No
9	6	2	3	1	-	-	No
10	7	2	6	3	-	-	No
11	3	2	1	1	-	-	No
12	5	1	2	1	-	-	Yes
13	4	1	1	0	3	1	Yes
14	7	1	6	3	5	2	Yes
15	2	0	1	0	3	1	Yes
16	5	1	1	1	-	-	No
17	6	1	4	1	-	-	Yes
18	6	1	2	0	-	-	Yes
19	8	1	3	2	-	-	No
20	7	2	9	4	-	-	No
21	8	2	2	1	-	-	No
22	9	1	5	0	-	-	Yes
23	5	2	0	0	2	1	No
24	3	1	0	0	2	1	Yes
25	3	1	0	0	-	-	No
26	8	5	-	-	-	-	No
27	9	2	5	1	0	0	Yes
28	7	2	2	1	-	-	Yes
29	4	1	1	0	-	-	No
30	5	1	3	2	-	-	No
31	0	0	0	0	-	-	No
32	4	1	-	-	-	-	No
33	3	2	4	2	-	-	No
34	6	2	-	-	-	-	No
35	6	1	0	0	0	0	Yes
36	5	2	4	1	3	1	Yes
37	7	2	2	1	-	-	No
38	6	2	2	1	2	1	No
39	6	1	2	2	-	-	No

40	5	1	2	1	1	1	No
41	6	2	2	1	-	-	No
42	9	2	4	3	-	-	No
43	9	2	3	1	2	1	No
44	4	2	-	-	-	-	No
45	5	1	2	1	3	2	Yes
46	8	2	3	2	-	-	No
47	6	4	3	1	-	-	No

Abbreviations: CTC, circulating tumor cells; PD-L1, programmed death-ligand 1; ORR, objective response rate.

Supplementary Fig. 1. Kaplan-Meier plots: (A) progression-free survival and (B) overall survival based on PD-L1 expression on CTCs at T1. Abbreviations: HR, hazard ratio; CI, confidence interval; CTCs, circulating tumor cells; PD-L1, programmed death-ligand 1.

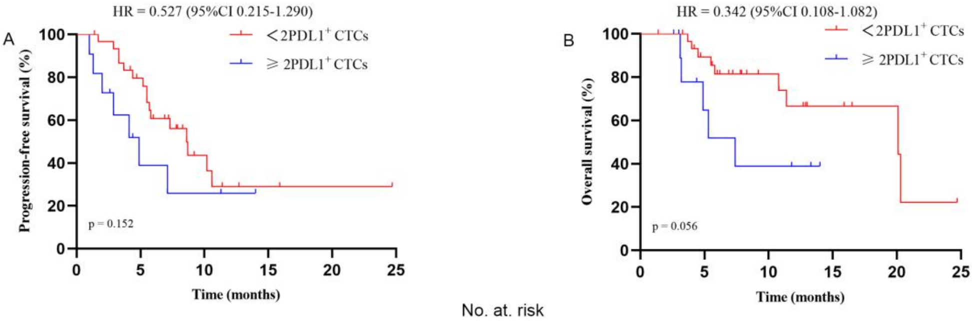

No. at. risk						
< 2 PD-L1+ CTCs	31	21	6	2	1	0
≥ 2 PD-L1+CTCs	11	3	2	0	0	0

No. at. risk						
< 2 PD-L1+ CTCs	31	23	11	5	3	0
≥ 2 PD-L1+ CTCs	11	5	3	0	0	0
